# Acute and Subacute Effects of Urban Air Pollution on Cardiopulmonary Emergencies and Mortality: Time Series Studies in Austrian Cities

**DOI:** 10.3390/ijerph10104728

**Published:** 2013-10-02

**Authors:** Manfred Neuberger, Hanns Moshammer, Daniel Rabczenko

**Affiliations:** 1 Institute of Environmental Health, Medical University of Vienna, A-1090 Kinderspitalgasse 15, Austria; E-Mail: manfred.neuberger@meduniwien.ac.at; 2 National Institute of Public Health – National Institute of Hygiene, PL-00-791 Warsaw, ul. Chocimska 24, Poland; E-Mail: daniel@pzh.gov.pl

**Keywords:** air pollution, particulate matter, nitrogen dioxide, emergencies, mortality, time series

## Abstract

Daily pollution data (collected in Graz over 16 years and in the Linz over 18 years) were used for time series studies (GAM and case-crossover) on the relationship with daily mortality (overall and specific causes of death). Diagnoses of patients who had been transported to hospitals in Linz were also available on a daily basis from eight years for time series analyses of cardiopulmonary emergencies. Increases in air pollutant levels over several days were followed by increases in mortality and the observed effects increased with the length of the exposure window considered, up to a maximum of 15 days. These mortality changes in Graz and Linz showed similar patterns like the ones found before in Vienna. A significant association of mortality could be demonstrated with NO_2_, PM_2.5_ and PM_10_ even in summer, when concentrations are lower and mainly related to motor traffic. Cardiorespiratory ambulance transports increased with NO_2_/PM_2.5_/PM_10_ by 2.0/6.1/1.7% per 10 µg/m^3^ on the same day. Monitoring of NO_2_ (related to motor traffic) and fine particulates at urban background stations predicts acute effects on cardiopulmonary emergencies and extended effects on cardiopulmonary mortality. Both components of urban air pollution are indicators of acute cardiopulmonary health risks, which need to be monitored and reduced, even below current standards.

## 1. Introduction

In most European cities PM_10_ and NO_2_ are used for surveillance of air quality, under the assumption that these air pollutants predict health effects best. NO_2_ is monitored as the surrogate of motor traffic exhaust, which is measured more easily than ultrafine particles. However, it is still unclear whether NO_2_ only modifies effects of PM_10_ on mortality [1] or if it is a key indicator of motor traffic and its health effects [2,3,4]. Recently European cities have in addition monitored PM_2.5_, because a limit was set for its annual concentration, however, a standard for daily PM_2.5_ concentration is still missing in Europe. Some health effects were found more closely related to NO_2_ [5,6] which is more variable with distance to road and correlated with black carbon and ultrafines, in particular from diesel soot. 

In multicentre studies similar associations between daily PM_10_ exposures and cardiorespiratory mortality were found in Europe and the United States [7,8]. Mortality increase with PM_10_, however, was higher in Canada [9], while in Europe most pronounced mortality effects were seen in warmer cities with higher NO_2_ and a larger NO_2_/PM_10_ ratio [10]. This pattern was less pronounced in the United States, where the proportion of diesel cars is lower. 

In Austria 55% of passenger cars and nearly all vans and trucks use diesel engines, because of the relatively low price of diesel fuel. In the capital Vienna a 10 µg/m^3^ change in PM_2.5_ was associated with a 2.6% change of daily mortality and a 10 µg/m^3^ change in NO_2_ with a 2.9% change of daily mortality, lagged 0–14 days. The increase of mortality [11] and hospital admissions [12,13] with PM and NO_2_ was seen in Vienna even in years without violation of EU standards (PM_10_ annual average 40 µg/m^3^, PM_10_ daily average 50 µg/m^3^ exceeded on not more than 35 days per year; NO_2_ hourly average 200 µg/m^3^ with maximal 18 exceedances per year). The present study investigates the associations of TSP, PM_10_, PM_2.5_, NO_2_, SO_2_ and O_3_ concentrations with daily mortality in a longer time series, in Graz, the 2nd largest and in Linz, the 3rd largest Austrian city, sharing similar populations and car fleets. The goal was to verify the results from Vienna in smaller cities and to find the best indicators for the prediction of short-term effects of urban air pollution on specific mortality. In addition to previously used generalized additive models (GAM), a case-crossover approach is used for the evaluation of air pollutant effects outside the heating season, to enable conclusions on motor traffic. As an additional outcome, cardiopulmonary emergencies transported to hospitals were available for a shorter time period in the city of Linz. These cases are also analysed in a case-crossover approach. 

## 2. Study Population and Methods

The city of Graz is situated south of the Alps in a semi alpine basin, with only very little industrial emissions. Nevertheless the EU standard for PM_10_ is exceeded regularly in winter, because of weak natural ventilation, long lasting inversions and stagnant weather conditions, when a locally aged aerosol forms from motor traffic and domestic heating [14,15]. Similar to Vienna, the city of Linz is located north of the Alps on the Danube, where air exchange is much better, resulting in lower ambient air pollution in winter, despite of a heavy industry conglomerate (iron, steel, chemistry). Like in Vienna SO_2_ had been reduced in both cities to low concentrations before 1990, leaving particulate matter (from traffic and heating) and NO_2_ (dominated by diesel powered vehicles) as the key pollutants [16], with data available after quality checks from 1990 to 2005 in Graz and from 1990 to 2007 in Linz. We used daily concentrations of pollutants (averaged over all available stations) as measures of exposure.

In Graz the number of inhabitants is only 261,540 (15% in comparison to Vienna) and in Linz 189,367 (11% in comparison to Vienna). From power calculations [17] we therefore had to choose longer time series than in Vienna, starting 1990. Methods used for monitoring of air pollutants and for modelling of missing data have been described earlier [12,15]. In short, time series from monitoring stations were used, which showed the highest correlation with all other urban background stations. Missing data were replaced by linear regression from the station with the second highest correlation to all other stations. In Graz exposure estimation was based on routine data of six stations, with five urban background stations monitoring TSP and NO_2_ since 1990. PM_10_ was monitored at these stations since 2000 and PM_2.5_ in 2000–2001 at one station only. Before 2000 PM_10_ was modelled from station specific relations to TSP and meteorological parameters. PM_2.5_ could not be modelled in Graz, because relations to measured values were available only for a single year and a single station. 

In Linz exposure estimation for the period 1990–2007 was based on six stations, four of them monitoring TSP and NO_2_ since 1990, PM_10_ since 2000 and one station monitoring PM_2.5_ in 2000–2001 and since 2005. Earlier missing data on PM_2.5_ were modelled from TSP. SO_2_, O_3_ and meteorological data were available for the complete period of observation in both cities. In Linz no sentinel data on influenza were available like in Vienna and Graz. In Graz sentinel data on influenza (registered 1990–2005 weekly and during epidemics daily) were used to adjust for the confounder of influenza epidemics, defined by an increase of incidence to >3,500 cases per week. Associations of air pollutants with hospital admissions (together with diagnoses at discharge) had been analysed in both cities before [12,13]. The present time series on daily mortality incidence in Graz and Linz used the same ICD-classes, overdispersed Poisson GAMs and lags (lag 0–1 day and polynomial distributed lags 0–7 and 0–14 days), which had been used in the Vienna study [11]. From death certificates the main or underlying cause of death is usually based on hospital diagnoses and high autopsy rates [18], ensuring a high diagnostic quality. Total mortality was analysed as well as deaths from specific causes, namely cardiovascular and respiratory diseases. In addition total mortality in the elderly (aged 65+) was investigated because this group might be more sensitive to effects of air pollution [19].

The model contained the following confounders: long-term trend and seasonality, day of the week, period of influenza epidemics and meteorological variables. The analysis started from building a base model without the air pollution variables. Thereby it was determined, what best described variability of daily mortality using all confounders. Day of the week and periods of influenza epidemics were modelled with dummy variables, other confounders were modelled using spline functions [20]. First the appropriate number of knots for the cubic regression spline representing long-term trend and seasonal variations was sought by minimising the sum of partial autocorrelation over 40 days [9]. Next meteorological factors were sequentially entered to the model: temperature, atmospheric pressure, humidity and changes of temperature and pressure between consecutive days. Different lags (0–3) and varying degrees of freedom were considered. Choice of lag structure was based on the Akaike Information Criterion [21]. 

Several aspects of air pollution effect on mortality were taken into account. The acute effect was modelled using linear and logarithmic function of mean of current and previous days. In addition the concentration-effect was further modelled as a spline function. Cumulative effects and a possible harvesting effect were investigated through a 3rd degree of polynomial distributed lag model. We also contemplated non-linear pollutant effects for the distributed lag model [22,23], but because the pollutants’ effects were fairly linear we abstained from these for the sake of interpretability. Difference in magnitude of air pollution effect by season (warm/cold) and level of other pollutants (high/low) were examined by creating appropriate dummy variables to be included in interaction terms. All computations were done using R 2.5.0 software applying the mgcv package for GAMs. For interactions between NO_2_ and PM at lag 0–1 we used models with interaction of PM_10_ and high NO_2_ and NO_2_ and high PM_10_, defining “high” as the median or above. 

In Linz morbidity data were also available from eight years: daily emergency transports to hospitals had been registered and diagnoses encoded in 2000–2007. This period in Linz was too short for stable results on mortality by GAM because of the small population and a high correlation between daily temperature and fine particulate concentration [17]. Therefore a case-crossover approach [24] was chosen, both for cardiopulmonary emergency transports and for cardiopulmonary and total deaths: exposures on case days were compared by conditional logistic regression with those on referent days occurring on a day with the most similar temperature (less than 1 °C difference) in the same season [25,26,27,28]. To eliminate time trends we chose a bi-directional approach and a time window of 7–21 days before and after the case (two referent days for each day of occurrence). This way temperature and season were eliminated as confounders and an overlap between periods of occurrence and referent periods was prevented. The time window of 7–21 days was narrow enough to find a day with equal temperature in the same season and it left a minimum distance of seven days to the occurrence day of cases at lag 0. This minimum distance was reduced to six days by analysing lags of one day, but it was not possible to analyse distributed lags of 0–7 days and 0–14 days without overlap of occurrence days and referent days. Therefore only short latencies could be analysed in the case-crossover study.

## 3. Results

Daily means of particulate matter and NO_2_ registered in Graz at five background stations and in Linz at three background stations and one roadside station are shown in Table 1. The distributions of the key pollutants PM_10_ and NO_2_ were similar, with higher median of NO_2_ in Linz (38 µg/m^3^) than in Graz (32 µg/m^3^), but lower 95^th^ percentile in Linz (62 µg/m^3^) than in Graz (68 µg/m^3^). The median/95^th^ percentile of PM10 was slightly higher in Linz (32/86 µg/m^3^) compared to Graz (29/73 µg/m^3^). The median/95^th^ percentile of TSP in Graz were 39/100 µg/m^3^ and the median/95^th^ percentile of PM_2.5_ in Linz were 19/49 µg/m^3^. 

SO_2_ concentrations were low most of the time (except for the first two winters observed in Graz): in Graz the median/95^th^ percentile was 7/30 µg/m^3^ and in Linz 5/19 µg/m^3^. Ozone was constantly higher during summer (especially during the last summer seasons observed), but had been monitored at elevated locations (Graz 348 m, 450 m and 661 m; Linz 265 m and 335 m), which were not representative for the exposures in domestic areas, where PM and NO_2_ had been monitored. In Graz the median/95^th^ percentile) was 49/96 µg/m^3^ and in Linz 41/86 µg/m^3^. 

**Table 1:** Descriptive statistics of pollution concentrations (µg/m^3^) and health effects (average per day).

**(A) NO_2_**	**Graz 1990–2005**	**Linz 1990–2007**	**Linz 2000–2007**	**Vienna 2000–2004**
Arithm. mean	35.7	39.4	34.6	23.0
Std. dev.	17.0	13.3	13.1	10.7
Median	31.9	38.4	32.8	21.3
5^th^ percentile	15.1	20.6	16.5	9.0
25^th^ percentile	24.1	30.1	26.0	14.9
75^th^ percentile	43.3	47.2	41.1	29.1
95^th^ percentile	68.5	62.2	58.0	43.0
**(B) TSP**	**Graz 1990–2005**	**Linz 1990–2007**	**Linz 2000–2007**	**Vienna 2000–2004**
Arithm. mean	45.8	-	-	30.6
Std. dev.	26.8	-	-	16.1
Median	38.8	-	-	27
5^th^ percentile	16.6	-	-	12
25^th^ percentile	27.4	-	-	19
75^th^ percentile	56.5	-	-	39
95^th^ percentile	100.2	-	-	61
**(C) PM10**	**Graz 1990–2005**	**Linz 1990–2007**	**Linz 2000–2007**	**Vienna 2000–2004**
Arithm. mean	34.0	38.1	33.31	30.2
Std. dev.	19.3	26.6	12.9	19.0
Median	29.1	31.9	28.2	25.6
5^th^ percentile	12.4	11.1	10.5	9
25^th^ percentile	20.7	20.4	18.4	16.8
75^th^ percentile	42.0	48.3	42.8	39.0
95^th^ percentile	73.4	85.8	73.8	66.8
**(D) PM2.5**	**Graz 1990–2005**	**Linz 1990–2007**	**Linz 2000–2007**	**Vienna 2000–2004**
Arithm. mean	-	22.4	20.5	16.3
Std. dev.	-	15.0	12.9	10.5
Median	-	18.8	17.1	13.7
5^th^ percentile	-	7.1	7.1	5
25^th^ percentile	-	12.5	11.7	8.9
75^th^ percentile	-	27.9	25.6	20.6
95^th^ percentile	-	49.3	45.9	36.2
**(E) End-points**	**Graz 1990–2005**	**Linz 1990–2007**	**Linz 2000–2007**	**Vienna 2000–2004**
Total deaths	6.9	5.6	5.4	43.9
Cardivasc.	3.6	2.6	2.4	23.2
Respiratory	0.3	0.4	0.4	2.3
Ambulance calls	-	-	15.7	-
Method	GAM	GAM	Case cross-over	GAM

Most deaths occurred on Mondays and least on Sundays. Mortality rates were highest in winter. When in sensitivity analysis degrees of freedom for the seasonal variation were increased the effect estimates for the pollutants were reduced, as was to be expected. But the impact of moderate changes in the degrees of freedom was only minimal (data not shown). Among the weather parameters temperature had the strongest effect both on mortality (in a typical u-shaped form) and on the estimates of the pollutants’ effects. Indeed in most of the full models SO_2_ seemed to be protective, which had been kept in the model as a sort of negative control despite of its low concentrations, for some endpoints and lags even significantly so, at least in Vienna and Linz. This implausible finding was removed or even reversed when temperature was not included in the models. This could eventually indicate some over-adjustment by temperature and was one of the reasons to stratify by temperature in the case-crossover models applied for the shorter time-series in Linz.

**Figure 1:**
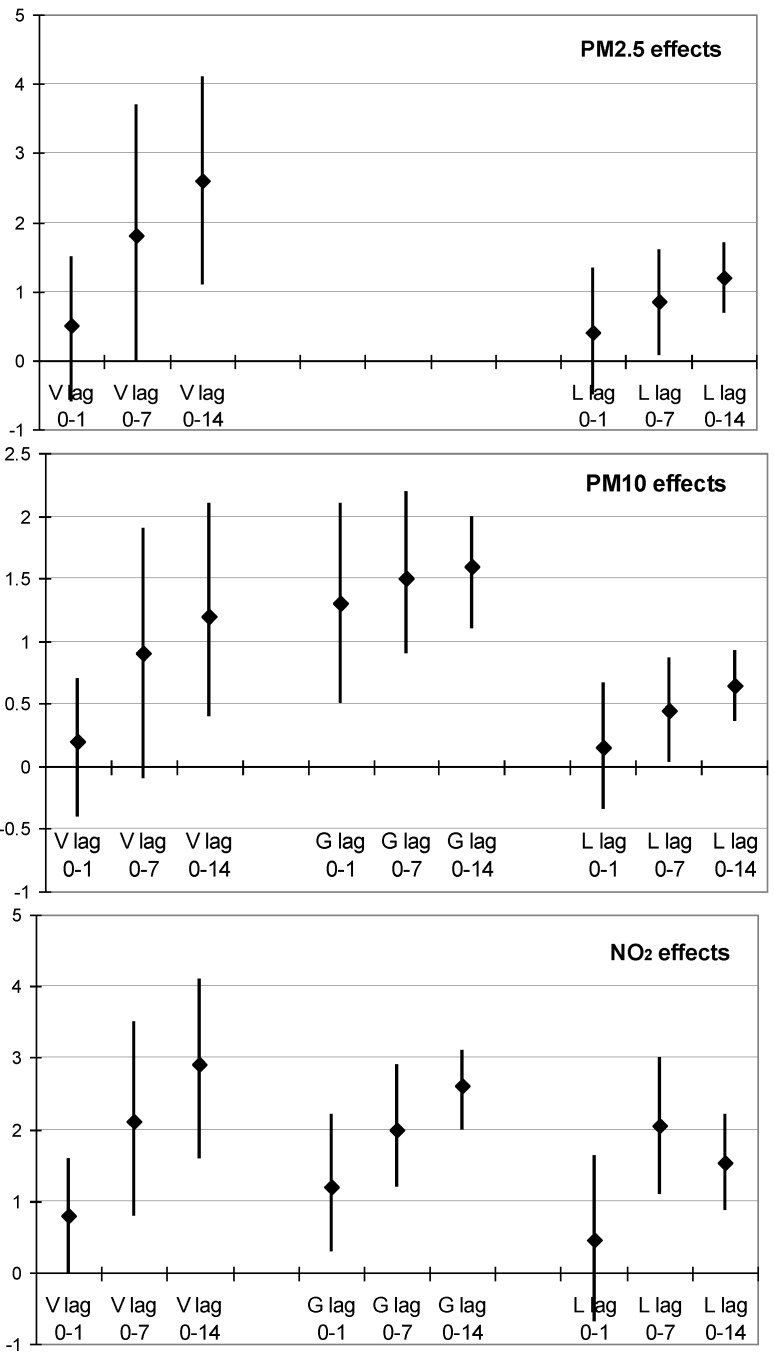
Per cent change in daily mortality, with 95% confidence intervals, in Graz (G) 1990–2005 and Linz (L) 1990–2007 per 10 µg/m^3^ increase in the respective pollutant measure over the preceding 2, 8, and 15 days (lag 0–1, lag 0–7, lag 0–14). Corresponding data [11] of Vienna (V) are shown for comparison.

**Figure S1:**
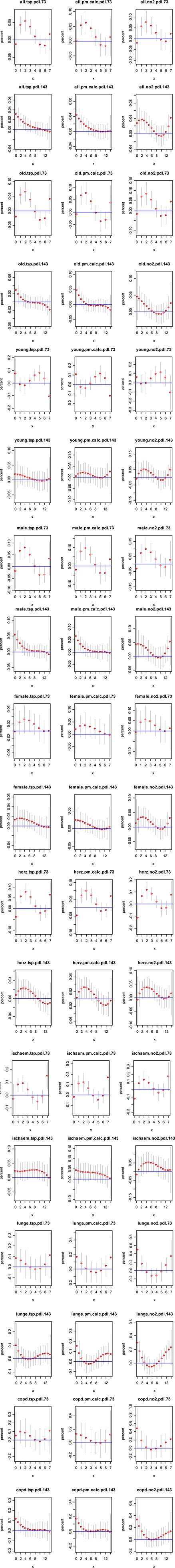
Temporal patterns of air pollution effects in Graz. The title of each graph indicates: cause of death, pollutant (pm.calc. indicating that PM_10_ was calculated from TSP), pdl (indicating that those are PDL models), and 73/143 (7 and 14 days lags, 3rd degree polynomial). X axis: day, Y axis: change (in%) of daily number of deaths associated with 10 mg/m^3^ increase.

Figure 1 and Supplemental Table S1 give effect estimates for daily overall mortality. Results for special age groups and selected causes of death are also presented in Supplemental Table S1. Results from the Vienna study [11] are included for comparison. The temporal distribution of effect estimates is exemplified for several mortality end-points in Graz in Supplemental Figure S1. Simpler models measuring the average exposure over 8 or 15 days instead of calculating the additive effects of 8 or 15 days distributed lags gave rather similar cumulative results (data not shown). 

In all three cities significant increases of mortality are seen with daily NO_2_ and fine particle mass. No significant changes were found for SO_2_ and O_3_ (data not shown). After an increase of NO_2_ or PM total mortality increases cumulatively with latency. Mortality increase was highest per µg/m^3^ for NO_2_. In Graz a mortality increase of 2.6% (3.2% in males and 2.1% in females) was found per 10 µg/m^3^ of NO_2_ for a lag of 0–14 days. Mortality increased less per µg/m^3^ of PM_10_ and TSP, but significantly. Also in Linz significant increases of mortality for lags 0–7 and 0–14 days were observed, related to NO_2_ (highest per µg/m^3^), PM_2.5_ and PM_10_ (lowest per µg/m^3^). Except for a higher effect of NO_2_ in Linz at lag 0–7 all pollutants show the highest effect at lag 0–14, indicating cumulative effects. Higher effects at longer lags are also seen in the results per interquartile range, with the highest estimate per IQR of NO_2_. None of the interaction terms between NO_2_ and PM_10_ was found to be significant in Graz and in Linz (data not shown). 

Missing data on PM_10_ and PM_2.5_ had been inferred from other measured pollutants for periods of several years and these estimates were pooled with measured data. We did sensitivity analyses to justify this procedure by investigating the interaction of PM effects with a dummy variable for the periods in which PM_10_ was calculated from TSP or PM_2.5_ from PM_10_. Results for the main effects of the pollutant remained unchanged (both coefficient value and its significance), both dummy variable and interaction term were statistically not significant.

The mortality increase was significant in both cities for males and females, for the elderly and for cardiopulmonary diseases. Also for specific causes of death the highest estimates were found at a lag of 0–14 days, with highest mean estimates for respiratory deaths (COPD in particular), followed by cardiovascular deaths, which reached 2.4% per 10 µg/m^3^ of NO_2_ in both cities at lag 0–14. In Graz at a lag of 0–7/0–14 days a mean increase of 2.9%/4.0% per 10 µg/m^3^ was estimated for deaths from ischemic heart diseases (IHD) related to NO_2_ and 3.7%/4.3% related to PM_10_. The corresponding figures for deaths from respiratory diseases (RD) were 8.1%/12.3% related to NO_2_ and 3.4%/5.1% related to PM_10_. Increases in Linz were lower, for RD in particular, with cardiovascular deaths more related to NO_2_. However, all results for deaths from IHD and RD were also in Linz significantly related to NO_2_ and PM_10_ at longer latencies (IHD at lag 0–7 and 0–14, and RD at lag 0–14). Daily deaths from gastrointestinal diseases (used for control in the time series) did not show any associations with air pollutant concentrations in Graz and Linz (data not shown). 

**Figure 2:**
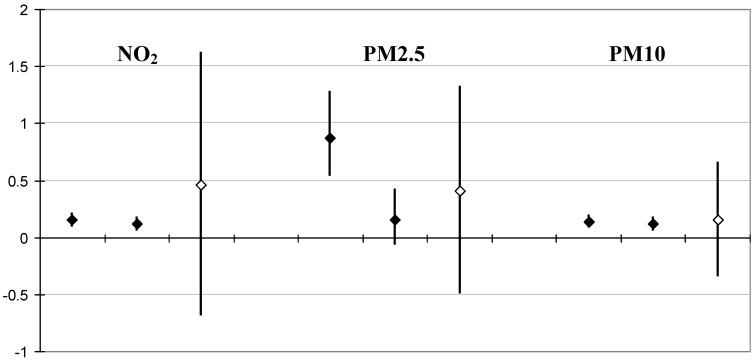
Effect estimates in Linz 2000–2007: Per cent change in daily mortality, with 95% confidence interval, per 10 µg/m^3^ increase in the respective pollutant measure. Full diamonds: Estimates from the years 2000–2007 on the same and on preceding day (lag 0 and lag 1), case-crossover model for the summer season (1st June to 31st August). For comparison Poisson GAM (open diamonds): estimates from the years 1990–2007 (full years) for the average of the same and preceding day (lag 0–1).

As a sensitivity analysis in Linz the period since 2000 (Figure 2) was studied separately when PM10 was measured directly and no longer had to be calculated from TSP [15]. The key pollutants measured in Linz from 2000 to 2007 showed a similar distribution of concentrations like in Vienna from 2000 to 2004. 

To exclude any effects from exposures to domestic heating (wood smoke), separate mortality analyses were run outside the heating seasons from June 1st to August 31st over eight years. Both approaches (case-crossover and GAM) estimate elevations of mortality after short latency (up to one day). Despite of the shorter time period available for the case-crossover analysis and the limitation to summer months, confidence intervals were found narrower than in the full period analysed by GAM. Per µg/m^3^ the effect on total mortality on the same day was highest for PM_2.5_. But the case-crossover model yields significant elevations of mortality also for NO_2_ and PM_10_ of the same and the preceding summer day.

**Table 2:** Per cent increase in ambulance calls (with 95% confidence intervals) related to a 10 µg/m^3^ increase in the respective pollutant measure. Linz (2000–2007).

	Lag 0 day	Lag 1 day	Lag 2 days
	**NO_2_**		
All cardiopulmonary	2.0 (0.8; 3.2)	−3.5 (−4.6; −2.4)	-
Cardiovascular	1.2 (−0.4; 2.8)	−4.2 (−5.7; −2.6)	−1.0 (−2.5; 0.6)
Respiratory	2.3 (−0.5; 5.1)	−3.2 (−5.9; −0.6)	0.3 (−2.4; 3.0)
Unclear cause	3.4 (1.2; 5.6)	−3.2 (−5.3; −1.0)	−0.1 (−2.3; 2.1)
	**PM_2.5_**		
All cardiopulmonary	6.1 (4.3; 7.8)	0.1 (−1.4; 1.7)	
Cardiovascular	7.1 (4.7; 9.4)	0.3 (−1.8; 2.4)	2.5 (0.3; 4.7)
Respiratory	2.1 (−2.1; 6.3)	−1.6; −5.2; 2.0)	1.0 (−2.7; 4.7)
Unclear cause	3.7 (0.4; 7.0)	0.4 (−2.5; 3.4)	3.4 (0.3; 6.4)
	**PM_10_**		
All cardiopulmonary	1.7 (1.0; 2.3)	0.0 (−0.6; 0.6)	
Cardiovascular	1.3 (0.9; 2.6)	−0.5 (−0.9; 0.7)	0.5 (0.1; 1.8)
Respiratory	2.7 (1.2; 4.2)	1.3 (−0.1; 2.7)	1.0 (−0.4; 2.5)
Unclear cause	1.6 (0.9; 3.3)	0.0 (−0.7; 1.6)	0.9 (0.2; 2.5)

In the case-crossover model significant elevations of cardiopulmonary emergencies (Table 2) were found related to NO_2_, PM_2.5_ and PM_10_, but not to O_3_. Per µg/m^3^ morbidity effects at lag 0 were most pronounced for PM_2.5_. No significant associations of cardiopulmonary emergencies or mortality were found with SO_2_ (data not shown). At lag 1 NO_2_, PM_2.5_ and PM_10_ appeared to be protective indicating a sort of “harvesting” effect.

## 4. Discussion

The association of cardiopulmonary morbidity and mortality with PM, NO_2_ and proximity to road are likely to share the same biological mechanisms: fine and ultrafine particles from combustion entering the lung, pulmonary reflexes over the autonomic nervous system affecting heart rate and arrhythmia, pulmonary inflammation, systemic inflammation from ultrafines entering the blood stream, endothelial dysfunction, reactive oxygen species, activation of leucocytes, platelets and other coagulation factors.

Health effects of air pollution are often classified into short- and long-term effects, although there is most probably a continuum of effects on the time scale, not yet completely understood. The results in Graz and Linz confirm earlier observations in Vienna [11], that prolonged mortality effects are higher than immediate effects, indicating that latent and cumulative effects outweigh harvesting (reduction of mortality after short increase due to premature deaths of most sensitive persons). Time series studies in Vienna had also found increases of respiratory hospital admissions related to PM_2.5_ [12] and of mortality from cardiovascular, cerebrovascular and respiratory diseases related to PM_10_, PM_2.5_ and NO_2_, most pronounced for a lag period of 15 days [12]. A time-series of daily hospital admissions in Vienna, Graz and Linz predicted the highest increases of cardiovascular admissions per µg/m^3^ for PM1, followed by PM_2.5_ and PM_10_, and also significant associations with daily NO_2_ concentrations were found [13]. The present study in Linz supplements, that also cardiopulmonary emergency transports in ambulance cars (preceding hospital admissions) rise quickly with daily concentrations of PM_2.5_, PM_10_ and NO_2_. In Vienna overall and cardiovascular mortality increased with latency and reached highest values per µg/m^3^ of NO_2_, followed by PM_2.5_. The same was found for overall mortality in Graz and Linz (Figure 1) and for cardiopulmonary mortality, indicating that legally binding limit values of daily PM_10_ might be less important than limitation of fine and ultrafine particles associated with NO_2_.

As an advantage of the time-series approach [29,30] the same population is used as exposed (on high pollution days) and as “control” (on low pollution days). Therefore no confounding by individual characteristics is probable. In contrast to cohort studies, time series are not confounded by risks spatially correlated to air pollution like traffic noise [31]. Daily means of urban air pollution are rather dependent on meteorological factors and day of the week (controlled in the present study) than on daily means of traffic noise, the socio-economic status or the daily dose of tobacco smoke (unrelated in time to ambient air pollution). 

Differences between the three largest Austrian cities have to be examined in cohort studies, but we think that the present study found more similarities than differences of results. Differences in attribution of underlying cause of death to pulmonary or cardiovascular disease in elderly persons could be influenced by local diagnostic and coding peculiarities; however, overall mortality is mainly influenced by the age distribution of the population, which was found to be very similar in Graz, Linz and Vienna [18]. Interactions of outdoor pollution with effects of active and passive smoking cannot be excluded; however, the same regulations apply for tobacco control in the three cities and smoking rates in 2006–2007 were similar [32]: Graz 20.9% (males 28.2%, females 14.2%) Linz 22.5% (males 23.2%, females 21.9%), Vienna 24.6% (males 30.3%, females 19.6%). A speculative explanation for the smaller effect of PM_10_ on mortality in Linz is the short distance of one of the stations in Linz to a tunnel portal of a busy road, where higher concentrations were measured than in domestic areas, possibly leading to an overestimation of mean exposure and an underestimation of the slope in the concentration-response relationship. 

In Linz a very quick rise of cardiopulmonary emergencies was observed with NO_2_, PM_2.5_ and PM_10_, even in summer (without heating) at levels below current standards. Together with the results on mortality this acute increase of cardiopulmonary morbidity indicates that urban air pollution is still a matter of concern and confirms the demand to lower PM_2.5_ standards and to introduce a limit value for the daily mean of PM_2.5_ in the European Union [33]. 

**Figure 3:**
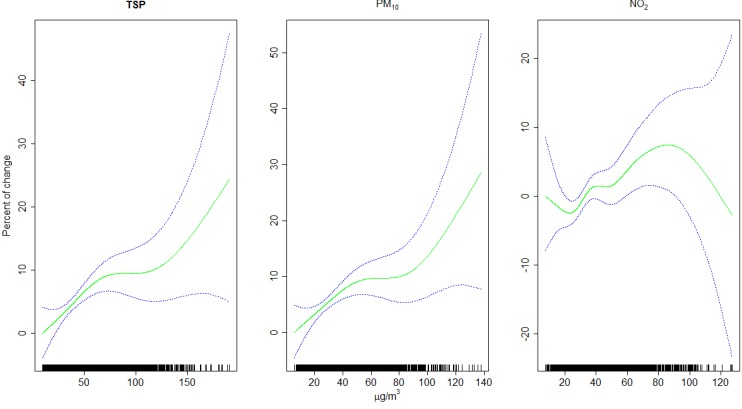
Per cent change in daily mortality, with 95% confidence intervals, in Graz (1990–2005), related to pollutants’ concentration over the same and the preceding day (lag 0–1). Degrees of freedom: 3.1 (TSP), 3.3 (PM10), 5.4 (NO_2_).

In Graz a significant increase of total mortality was seen already at short latencies with TSP and PM10, below a daily PM_10_ concentration of 50 µg/m^3^, which is the EU limit for the daily mean. This increase of mortality was steep at concentrations below the current standard of PM_10_ and flattened above (Figure 3). At very high concentrations we observed a second increase; however, the estimate from rare events is unreliable, as can be seen from the widening confidence intervals at high concentrations. What could be concluded from the concentration-response function is the absence of a threshold, as found by other investigators [8,34], but questions also the present regulations: Figure 3 seems to contradict the current methods of surveillance of daily PM_10_ concentrations with reporting of the number of exceedances of 50 µg/m^3^ per year: From short-term mortality effects in Graz it seems to be of low relevance if the PM_10_ concentration is a little below or above the standard of 50 µg/m^3^. On the other hand considerable health benefits are expected from reductions of pollutants also at locations where and at times when concentrations are very much below the current standard. For NO_2_ the concentration-response function shows an increase of short-term mortality in the concentration range of about 25 to 90 µg/m^3^. The estimate gets unreliable at very low and especially at very high concentrations, but from Figure 3 no indication for a threshold higher than 25–30 µg/m^3^ can be seen. On the other hand, also these effects level off, when daily means of about 35 µg/m^3^ are exceeded, but this flattening of the curve is only seen up to about 50 µg/m^3^, which is frequently exceeded due to motor traffic at urban background stations in domestic areas. Most pronounced are the prolonged effects of NO_2_ on mortality, which can hardly be explained by the gas itself. More likely NO_2_ is a surrogate for ultrafine particles from motor traffic and diesel exhaust in particular. Therefore this study supports demands for a better surveillance of traffic-related pollutants. Emission concentrations of ultrafine particles are regulated already in the EU [35]. 

The results of the present study contribute to the “suggestive evidence of a causal relationship” between exposure to traffic-related air pollution and total (mainly cardiovascular) mortality [8]: NO_2_ indicates combustion and motor traffic. The contribution of motor traffic to PM increases with decreasing particle size. Therefore the effects on mortality, most pronounced per µg/m^3^ of NO_2_ and increasing with decreasing particle size of PM in Graz, Linz and Vienna, indicate health effects of motor traffic. In all three cities NO_2_ is mainly of local origin, also in summer [36]. By contrast, in Linz and Vienna about 60% of PM_10_ is imported [37], while in Graz PM_10_ is also mainly of local origin, with a high traffic share [38]. Even in Linz the contribution of the industry to NO_2_ exposure of the population is small and in Graz it is negligible. In contrast to studies in more industrialized areas [39], we therefore think that the results on NO_2_-related health effects in this study allow conclusions on health effects of motor traffic and diesel engines in particular. Up to now studies on proximity to roads [40,41] are unable to say, which part of health effects are related to the irritant gas NO_2_ itself, to accompanying ultrafine particles or to traffic noise, which had also been suggested as a risk factor for cardiovascular diseases [42], or to other spatially correlated risk factors. For PM also Goodman *et al.* [43] observed that extended effects of air pollution exceed its acute effects. To our knowledge there are no studies on subacute mortality and NO_2_, however, in cohort studies higher cumulative effects had been found after much longer latencies for both PM [39,40] and NO_2_ [3,4].

Data on PM_10_ were not available before 2000 and had to be modelled from TSP and meteorology. Nevertheless PM_10_ effects were found to exceed those of TSP, as expected. PM_2.5_ had been monitored in Graz and Linz in 2000–2001 and in Linz since 2005. Despite of the necessity to model the PM_2.5_ concentrations in Linz from PM_10_ in 2002–2004 and from TSP in earlier years, PM_2.5_ was found to predict cardiopulmonary emergencies and mortality (Figure 1) very well.

Separate time series over the summer months did not reveal a significant effect of ozone on mortality, but significant elevations related to NO_2_, PM_2.5_ and PM_10_. This underlines the importance of motor traffic exhaust for acute effects on mortality in urban environments. The absence of mortality effects related to ozone was also observed in Vienna [11] and might be due to the sampling strategy in all three cities, which was different from other pollutants: Ozone was not monitored in densely populated areas like PM_10_, NO_2_ and SO_2_, but at elevations and downwind of urban precursor emissions, where few people lived. We cannot exclude small effects of O_3_ on respiratory mortality in summer months, detectable only in larger cities. A longer time series from Vienna with sufficient power could recently show ozone effects on mortality [43] using data of a central station. 

SO_2_ effects were not seen in Graz, Linz and Vienna during the available person-years of observation, however, already before the begin of this study, during the 1980s, Austria had achieved the highest SO_2_ reduction among the signatory states of the Helsinki Protocol [12] and these low concentrations over most of the observation period, could explain the lack of association of SO_2_ with mortality.

For Vienna emissions of NO_2_ were estimated to decrease [44] from 2010 to 2020 by 513 tons only (road traffic NO_2_ from 1,590 to 1,090 tons per year). During the same decade a reduction of PM_10_ by 240 tons was estimated (road traffic from 770 to 570 tons per year). This does not give hope for a disappearance of related health effects in the near future.

## 5. Conclusions

The concentrations of NO_2_ and small particles predicted health effects of urban air pollution in the three largest cities of Austria very well. Therefore we see no necessity at present to supplement surveillance by other indicators of combustion and motor traffic such as black carbon [45,46] or ultrafine particles [47]. Changing motor technology (direct injection, *etc.*) and fuel-additives, however, could possibly require additional indicators of ambient air pollution in the future. 

From the results in Graz and Linz we conclude, that also smaller cities face acute health effects of air pollution from local motor traffic. This study supports the conclusion [12,33] that more stringent standards need to be adopted in Europe to protect public health from ambient PM_2.5_ and it adds, that also further reductions of NO_2_ might be necessary on local, national and EU level, accompanied by epidemiological studies.

## Supplemental Materials

**Table S1:** Lag-structure and degrees of freedom in the models. (**A**) Per cent increase in mortality (with 95% confidence intervals) related to a 10 µg/m^3^ increase in pollution—Vienna. (**B**) Per cent increase in mortality (with 95% confidence intervals) related to a 10 µg/m^3^ increase in pollution—Graz. (**C**) Per cent increase in mortality (with 95% confidence intervals) related to a 10 µg/m^3^ increase in pollution—Linz (1990–2007).

**(A) Vienna**	**Lag 0–1 day**	**Lag 0–7 days**	**Lag 0–14 days**
	**NO_2_**		
All deaths	0.8 (0.0; 1.6)	2.1 (0.8; 3.5)	2.9 (1.6; 4.1)
Cardiovascular	0.7 (−0.4; 1.8)	3.1 (1.2; 5.0)	4.6 (2.9; 6.3)
Ischemic heart	0.4 (−1.2; 1.9)	2.8 (0.3; 5.3)	4.5 (2.4; 6.6)
Other heart	1.6 (−1.1; 4.5)	4.6 (0.4; 9.1)	3.9 (0.4; 7.6)
Cerebrovascular	1.0 (−1.7; 3.9)	2.4 (−1.8; 6.7)	4.4 (0.8; 8.2)
Respiratory	2.3 (−0.6; 5.2)	5.7 (1.1; 10.6)	6.7 (2.7; 10.8)
COPD	1.9 (−2.4; 6.4)	9.9 (3.0; 17.3)	8.9 (3.0; 15.2)
	**TSP**		
All deaths	0.3 (−0.3; 0.9)	0.9 (−0.0; 1.9)	0.8 (0.0; 1.6)
Cardiovascular	0.3 (−0.5; 1.2)	1.6 (0.3; 3.0)	1.7 (0.7; 2.8)
Ischemic heart	0.8 (−0.4; 2.1)	2.6 (0.8; 4.5)	3.4 (1.9; 4.9)
Other heart	0.3 (−1.8; 2.4)	0.9 (−2.2; 4.1)	−1.3 (−3.7; 1.2)
Cerebrovascular	0.1 (−2.0; 2.3)	0.9 (−2.3; 4.1)	1.7 (−0.9; 4.3)
Respiratory	2.3 (0.0; 4.7)	3.7 (0.1; 7.4)	2.7 (−0.1; 5.6)
COPD	3.6 (0.2; 7.1)	8.5 (3.1; 14.2)	5.0 (0.7; 9.4)
	**PM_2.5_**		
All deaths	0.5 (−0.6; 1.5)	1.8 (−0.0; 3.7)	2.6 (1.1; 4.1)
Cardiovascular	0.4 (−1.0; 1.8)	2.7 (0.3; 5.2)	3.8 (1.9; 5.8)
Ischemic heart	1.0 (−1.0; 3.1)	3.8 (0.6; 7.1)	5.5 (3.0; 8.1)
Other heart	0.9 (−2.6; 4.6)	3.0 (−2.2; 8.5)	1.5 (−2.5; 5.7)
Cerebrovascular	0.4 (−3.2; 4.1)	2.7 (−2.7; 8.4)	4.9 (0.7; 9.3)
Respiratory	3.9 (0.1; 7.8)	7.0 (0.9; 13.4)	6.4 (1.9; 11.2)
COPD	6.4 (0.6; 12.5)	14.0 (4.9; 24.0)	9.0 (1.9; 16.6)
	**PM_10_**		
All deaths	0.2 (−0.4; 0.7)	0.9 (−0.1; 1.9)	1.2 (0.4; 2.1)
Cardiovascular	−0.2 (−0.6; 1.0)	1.4 (0.1; 2.8)	2.0 (0.9; 3.1)
Ischemic heart	0.4 (−0.7; 1.6)	2.1 (0.4; 3.9)	3.2 (1.8; 4.6)
Other heart	0.4 (−1.5; 2.4)	1.3 (−1.6; 4.3)	−0.1 (−2.4; 2.2)
Cerebrovascular	0.3 (−1.6; 2.4)	1.4 (−1.6; 4.4)	2.6 (0.3; 5.0)
Respiratory	2.1 (0.0; 4.2)	3.5 (0.1; 6.9)	3.0 (0.5; 5.5)
COPD	3.5 (0.4; 6.7)	7.9 (3.0; 12.9)	5.1 (1.3; 9.1)
**(B) Graz**	**Lag 0–1 day**	**Lag 0–7 days**	**Lag 0–14 days**
	**NO_2_**		
All deaths	1.2 (0.3; 2.2)	2.0 (1.2; 2.9)	2.6 (2.0; 3.1)
65+	1.6 (−0.6; 3.8)	2.0 (1.1; 2.9)	1.9 (1.3; 2.5)
Before 65	1.2 (0.1; 2.3)	2.6 (0.7; 4.5)	2.5 (1.2; 3.9)
Male	1.3 (0; 2.7)	2.4 (1.2; 3.7)	3.2 (2.3; 4.1)
Female	1.1 (−0.2; 2.4)	1.8 (0.7; 2.9)	2.1 (1.3; 2.9)
Cardiovascular	0.3 (−1.1; 1.7)	1.9 (0.7; 3.0)	2.4 (1.6; 3.2)
Ischemic heart	0.5 (−1.6; 2.7)	2.9 (1.1; 4.8)	4.0 (2.7; 5.4)
COPD	7.8 (2.2; 13.7)	10.8 (5.9; 16.0)	13.9 (10.3; 17.5)
Respiratory	6.2 (1.7; 11.0)	8.1 (4.1; 12.2)	12.3 (9.5; 15.2)
	**TSP**		
All deaths	0.9 (0.4; 1.5)	1.2 (0.7; 1.7)	1.2 (0.9; 1.5)
65+	1.1 (−0.1; 2.3)	1.2 (0.7; 1.7)	0.1 (−0.3; 0.4)
Before 65	0.9 (0.3; 1.5)	1.4 (0.3; 2.4)	0.9 (0.2; 1.6)
Male	1.1 (0.3; 1.9)	1.5 (0.8; 2.2)	1.5 (1.1; 2.0)
Female	0.8 (0; 1.5)	1.1 (0.5; 1.7)	1.1 (0.6; 1.5)
Cardiovascular	1.2 (0.5; 2.0)	1.9 (1.2; 2.5)	0.9 (0.4; 1.3)
Ischemic heart	1.3 (0.1; 2.6)	2.6 (1.5; 3.6)	3.1 (2.4; 3.8)
COPD	2.9 (−0.1; 6.0)	3.7 (0.9; 6.4)	4.0 (2.1; 5.9)
Respiratory	1.9 (−0.6; 4.5)	2.4 (0.2; 4.7)	3.7 (2.2; 5.3)
	**PM_10_**		
All deaths	1.3 (0.5; 2.1)	1.5 (0.9; 2.2)	1.6 (1.1; 2.0)
65+	1.3 (−0.4; 3.1)	1.5 (0.8; 2.2)	0.3 (−0,1; 0.8)
Before 65	1.3 (0.4; 2.2)	1.8 (0.4; 3.3)	1.5 (0.5; 2.5)
Male	1.4 (0.2; 2.5)	1.8 (0.8; 2.8)	2.0 (1.4; 2.7)
Female	1.2 (0.1; 2.3)	1.5 (0.6; 2.3)	1.3 (0.8; 1.9)
Cardiovascular	1.6 (0.6; 2.7)	2.5 (1.6; 3.4)	1.0 (0.4; 1.7)
Ischemic heart	2.0 (0.2; 3.8)	3.7 (2.3; 5.2)	4.3 (3.2; 5.3)
COPD	3.5 (−0.8; 7.9)	4.3 (0.6; 8.2)	4.6 (2.1; 7.2)
Respiratory	2.8 (−0.8; 6.4)	3.4 (0.4; 6.5)	5.6 (3.5; 7.8)
(C) Linz	Lag 0–1 day	Lag 0–7 days	Lag 0–14 days
	**NO_2_**		
All deaths	0.5 (−0.7; 1.6)	2.0 (1.1; 3.0)	1.5 (0.9; 2.2)
Cardiovascular	0.1 (−1.5; 1.7)	2.3 (0.9; 3.7)	2.4 (1.5; 3.4)
Ischemic heart	1.0 (−1.4; 3.4)	3.8 (1.7; 5.9)	2.5 (1.0; 3.9)
COPD	1.1 (−4.1; 6.7)	1.1 (−3.6; 6.0)	1.5 (0.8; 2.3)
Respiratory	1.9 (−2.3; 6.3)	2.4 (−1.1; 6.0)	2.5 (1.5; 3.5)
65 +	0.4 (−0.9; 1.7)	2.0 (0.9; 3.1)	1.5 (0.7; 2.2)
Male	1.2 (−0.3; 2.7)	2.0 (0.6; 3.4)	1.8 (0.8; 2.8)
Female	0.0 (1.5; 1.5)	1.9 (0.6; 3.1)	1.9 (1.0; 2.8)
	**PM_2.5_**		
All deaths	0.4 (−0.5; 1.3)	0.8 (0.1; 1.6)	1.2 (0.7; 1.7)
Cardiovascular	0.5 (−0.7; 5.9)	1.8 (0.7; 2.9)	3.3 (2.5; 4.1)
Ischemic heart	0.6 (−1.4; 2.6)	2.1 (0.4; 3.8)	2.0 (0.9; 3.2)
COPD	1.3 (−2.9; 5.6)	0.3 (−3.5; 4.4)	1.3 (0.8; 1.9)
Respiratory	2.5 (−0.7; 5.9)	2.1 (−0.8; 5.0)	3.6 (2.8; 4.3)
65 +	0.7 (−0.4; 1.7)	1.2 (0.3; 2.0)	1.4 (0.9; 2.0)
Male	0.4 (−0.8; 1.6)	0.5 (−0.6; 1.6)	0.8 (0.1; 1.6)
Female	0.0 (−1.2; 1.2)	0.6 (−0.4; 1.7)	0.7 (0.0; 1.4)
	**PM_10_**		
All deaths	0.2 (−0.3; 0.7)	0.4 (0.0; 0.9)	0.6 (0.4; 0.9)
Cardiovascular	0.3 (−0.4; 1.0)	1.1 (0.5; 1.7)	2.0 (1.5; 2.4)
Ischemic heart	0.4 (−0.7; 1.5)	1.3 (0.4; 2.3)	1.3 (0.7; 2.0)
COPD	0.4 (−2.0; 2.8)	0.0 (−2.2; 2.2)	0.8 (0.5; 1.1)
Respiratory	1.1 (−0.8; 2.9)	1.0 (−0.6; 2.6)	2.1 (1.7; 2.5)
65 +	0.3 (−0.3; 0.8)	0.7 (0.2; 1.1)	0.9 (0.6; 1.2)
Male	0.1 (−0.6; 0.8)	0.2 (−0.4; 0.8)	0.3 (−0.1; 0.7)
Female	0.1 (−0.5; 0.8)	0.6 (0.0; 1.2)	0.7 (0.4; 1.1)

## References

[1] Samoli E., Analitis A., Touloumi G., Schwartz J., Anderson H.R., Sunyer J., Bisanti L., Zmirou D., Vonk J.M., Pekkanen J. (2005). Estimating the exposure-response relationships between particulate matter and mortality within the APHEA multicity project. Environ. Health Perspect..

[2] Lambrechtsen J., Gerke O., Egstrup K., Sand N.P., Nørgaard B.L., Petersen H., Mickley H., Diederichsen A.C. (2012). The relation between coronary artery calcification in asymptomatic subjects and both traditional risk factors and living in the city centre: A DanRisk substudy. J. Intern. Med..

[3] Naess O., Nafstad P., Aamodt G., Claussen B., Rosland P. (2007). Relation between concentration of air pollution and cause-specific mortality: Four-year exposures to nitrogen dioxide and particulate matter pollutants in 470 neighborhoods in Oslo, Norway. Am. J. Epidemiol..

[4] Nafstad P., Haheim L.L., Wisloff T., Gram F., Oftedal B., Holme I., Hjermann I., Leren P. (2004). Urban air pollution and mortality in a cohort of Norwegian Men. Environ. Health Perspect..

[5] Chen R., Samoli E., Wong C.M., Huang W., Wang Z., Chen B., Kan H., CAPES Collaborative Group (2012). Associations between short-term exposure to nitrogen dioxide and mortality in 17 Chinese cities: The China Air Pollution and Health Effects Study (CAPES). Environ. Int..

[6] Lenters V., Uiterwaal C.S., Beelen R., Bots M.L., Fischer P., Brunekreef B., Hoek G. (2010). Long-term exposure to air pollution and vascular damage in young adults. Epidemiology.

[7] HEI (Health Effects Institute) (2003). Revised Analyses of Time-Series Studies of Air Pollution and Health.

[8] HEI (Health Effects Institute) (2010). Traffic-Related Air Pollution. A Critical Review of the Literature on Emissions, Exposure, and Health Effects.

[9] Samoli E., Peng R., Ramsay T., Pipikou M., Touloumi G., Dominici F., Burnett R., Cohen A., Krewski D., Samet J., Katsouyanni K. (2008). Acute effects of ambient particulate matter on mortality in Europe and North America: Results from the APHENA study. Environ. Health Perspect..

[10] Samoli E., Touloumi G., Zanobetti A., Le Tertre A., Shindler C., Atkinson R., Vonk J., Rossi G., Saez M., Rabczenko D. (2003). Investigating the dose-response relation between air pollution and total mortality in the APHEA-2 multicity project. Occup. Environ. Med..

[11] Neuberger M., Rabczenko D., Moshammer H. (2007). Extended effects of air pollution on cardiopulmonary mortality in Vienna. Atmos. Environ..

[12] Neuberger M., Schimek M.G., Horak F., Moshammer H., Kundi M., Frischer T.,  Gomiscek B., Puxbaum H., Hauck H., AUPHEP-Team (2004). Acute effects of particulate matter on respiratory diseases, symptoms and functions. Epidemiological results of AUPHEP. Atmos. Environ..

[13] Neuberger M., Schimek M.G., Moshammer H., Hauck H., Kofler W. (2008). Fine particulates and hospital admissions in Graz, Linz, and Vienna. Atemw. Lungenkrht..

[14] Almbauer R., Pucher K., Sturm P.J. (2005). Air quality modeling for the city of Graz. Meteorol. Atmos. Environ..

[15] Hauck H., Berner A., Frischer T., Gomiscek B., Kundi M., Neuberger M., Puxbaum H., Preining O., AUPHEP-Team (2004). Austrian project on health effects of particulates—General overview. Atmos. Environ..

[16] Spangl W., Nagl C. (2011). Annual Report of Air Quality Measurements in Austria 2010.

[17] Winquist A., Klein M., Tolbert P., Sarnat S. (2012). Power estimation using simulations for air pollution time-series studies. Environ. Health.

[18] Statistik Austria (2009). Demographic Yearbook 2008.

[19] Cakmak S., Dales R.E., Rubio M.A., Vidal C.B. (2011). The risk of dying on days of higher air pollution among the socially disadvantaged elderly. Environ. Res..

[20] Baccini M., Biggeri A., Lagazio C., Lertxundi A., Saez M. (2007). Parametric and semi-parametric approaches in the analysis of short-term effects of air pollution on health. Comput. Statist. Data Anal..

[21] Akaike H., Petrov B.N., Csaki B.F. (1973). Information theory as an extension of the maximum likelihood principle. Proceedings of the Second International Symposium on Information Theory.

[22] Gasparrini A., Armstrong B. (2010). Time series analysis on the health effects of temperature: Advancements and limitations. Environ. Res..

[23] Goldberg M.S., Gasparrini A., Armstrong B., Valois M.-F. (2011). The short-term influence of temperature on daily mortality in the temperate climate of Montreal, Canada. Environ. Res..

[24] Carracedo-Martínez E., Taracido M., Tobias A., Saez M., Figueiras A. (2010). Case-Crossover Analysis of Air Pollution Health Effects: A Systematic Review of Methodology and Application. Environ. Health Perspect..

[25] Schwartz J. (2004). The effects of particulate air pollution on daily deaths: A multi-city case crossover analysis. Occup. Environ. Med..

[26] Schwartz J. (2005). How sensitive is the association between ozone and daily deaths to control for temperature?. Am. J. Respir. Crit. Care Med..

[27] Zanobetti A., Schwartz J. (2006). Air pollution and emergency admissions in Boston, MA. J. Epidemiol. Community Health.

[28] Scheers H., Mwalili S.M., Faes C., Fierens F., Nemery B., Nawrot T.S. (2011). Does air pollution trigger infant mortality in Western Europe? A case-crossover study. Environ. Health Perspect..

[29] Zheng S., Wang M., Wang S., Tao Y., Shang K. (2013). Short-Term Effects of Gaseous Pollutants and Particulate Matter on Daily Hospital Admissions for Cardio-Cerebrovascular Disease in Lanzhou: Evidence from a Heavily Polluted City in China. Int. J. Environ. Res. Public Health.

[30] Zhang F., Li L., Krafft T., Lv J., Wang W., Pei D. (2011). Study on the Association between Ambient Air Pollution and Daily Cardiovascular and Respiratory Mortality in an Urban District of Beijing. Int. J. Environ. Res. Public Health.

[31] Allen R.W., Davies H., Cohen M.A., Mallach G., Kaufman J.D., Adar S.D. (2009). The spatial relationship between traffic-generated air pollution and noise in 2 US cities. Env. Res..

[32] Klimont J., Kytir J., Leitner B. (2007). Austrian Health Survey 2006/2007.

[33] Ballester F., Medina S., Boldo E., Goodman P., Neuberger M., Iñiguez C., Künzli N. (2008). Reducing ambient levels of fine particulates could substantially improve health: A mortality impact assessment for 26 European cities. J. Epidemiol. Community Health.

[34] WHO (World Health Organisation) (2006). Air Quality Guidelines for Particulate Matter, Ozone, Nitrogen Dioxide and Sulfur Dioxide—Global Update 2005—Summary of Risk Assessment.

[35] Giechaskiel B., Mamakos A., Andersson J., Dilara P., Martini G., Schindler W., Bergmann A. (2012). Measurement of Automotive Nonvolatile Particle Number Emissions within the European Legislative Framework: A Review. Aerosol Sci. Technol..

[36] Kurz C., Sturm P. (2011). Examination of the NO_2_ and PM10 Contamination in the Urban Area of Linz.

[37] Kreindl J., Hager W. (2011). Air Quality Data in 2010.

[38] Öttl D. (2008). Modelling. Primary PM10 Concentrations for the City of Graz, Austria.

[39] Gehring U., Heinrich J., Kramer U., Grote V., Hochadel M., Sugiri D., Kraft M., Rauchfuss K., Eberwein H.G., Wichmann H.E. (2006). Long-Term Exposure to Ambient Air Pollution and Cardiopulmonary Mortality in Women. Epidemiology.

[40] Hoek G., Brunekreef B., Goldbohm S., Fischer P., van den Brandt P.A. (2002). Association between mortality and indicators of traffic-related air pollution in the Netherlands: A cohort study. Lancet.

[41] Rosenbloom J.I., Wilker E.H., Mukamal K.J., Schwartz J., Mittleman M.J. (2012). Residential proximity to major roadway and 10-Year all-cause mortality after myocardial infarction. Circulation.

[42] Bluhm G., Eriksson C. (2011). Cardiovascular effects of environmental noise: Research in Sweden. Noise Health.

[43] Goodman P.G., Dockery D.W., Clancy L. (2004). Cause-specific mortality and the extended effects of particulate pollution and temperature exposure. Environ. Health Perspect..

[44] Moshammer H., Hutter H.-P., Kundi M. (2013). Which metric of ambient ozone to predict daily mortality?. Atmos. Environ..

[45] Kurz C., Orthofer R., Sturm P., Kaiser A., Uhrner U., Reifeltshammer R. Projection of the air quality in Vienna between 2005 and 2020 for NO_2_ and PM10. Proceedings of the 8th International Conference on Air Quality.

[46] Dons E., Panis L.I., Van Poppel M., Theunis J., Wets G. (2012). Personal exposure to Black Carbon in transport microenvironments. Atmos. Environ..

[47] Janssen N.A.H., Gerlofs-Nijland M.E., Lanki T., Salonen R.O., Cassee F., Hoek G. (2012). Health Effects of Black Carbon.

[48] Wang Y., Hopke P.K., Chalupa D.C., Utell M.J. (2011). Long-term study of urban ultrafine particles and other pollutants. Atmos. Environ..

